# Increasing the expression of microRNA-126-5p in the temporal muscle can promote angiogenesis in the chronically ischemic brains of rats subjected to two-vessel occlusion plus encephalo-myo-synangiosis

**DOI:** 10.18632/aging.103431

**Published:** 2020-07-09

**Authors:** Chuan Chen, Cong Ling, Jin Gong, Chao Li, Liying Zhang, Shuangqi Gao, Zhangyu Li, Tengchao Huang, Hui Wang, Ying Guo

**Affiliations:** 1 Department of Neurosurgery, Third Affiliated Hospital of Sun Yat-Sen University, Guangzhou 510630, Guangdong, PR China; 2 Department of Rehabilitation, Third Affiliated Hospital of Sun Yat-Sen University, Guangzhou 510630, Guangdong, PR China

**Keywords:** moyamoya disease, encephalo-myo-synangiosis, microRNA-126-5p, endothelial cell proliferation, angiogenesis

## Abstract

Background: miR-126-5p plays an important role in promoting endothelial cell (EC) proliferation. We thus explored whether miR-126-5p can promote EC proliferation and angiogenesis in chronically ischemic brains (CIBs).

Results: Improved revascularization in moyamoya patients was correlated with upregulated miR-126-5p expression in the TM and DM. In vitro experiments showed that miR-126-5p promoted EC proliferation through the PI3K/Akt pathway. CIBs from the agomir group exhibited significantly higher p-Akt, VEGF, CD31 and eNOS expression compared with the control CIBs. The ICBP and the RCF were significantly better in the agomir compared with the control group.

Conclusion: Increasing miR-126-5p expression in the TM can promote EC proliferation and angiogenesis in CIBs of 2VO+EMS rats through the PI3K/Akt pathway.

Methods: We assessed the correlation between revascularization and miR-126-5p expression in the temporal muscle (TM) and dura mater (DM) of moyamoya patients. The effect of miR-126-5p on EC proliferation and downstream signaling pathways was explored in vitro. We established an animal model of two-vessel occlusion plus encephalo-myo-synangiosis (2VO+EMS), transfected the TM with miR-126-5p agomir/antagomir, compared the expression of miR-126-5p and relevant downstream cytokines in brain tissue among different groups, and investigated the improvement in cerebral blood perfusion (ICBP) and the recovery of cognitive function (RCF).

## INTRODUCTION

Moyamoya disease is a chronic cerebrovascular disorder characterized by progressive stenosis and occlusion of the intracranial large arteries [1]. As patients with moyamoya disease age, the chances of hemorrhagic and ischemic stroke gradually increase [2]. Indirect revascularization surgeries, such as encephalo-myo-synangiosis (EMS), which can improve cerebral blood perfusion (CBP), are routine surgeries for the treatment of moyamoya disease [3]. However, nearly half of adult moyamoya patients unfortunately show poor potential for angiogenesis. For this subset of patients, ischemic brain tissues do not form adequate anastomosis with capillary-rich tissues such as the temporal muscle (TM) or dura mater (DM) after revascularization surgery, which prevents the effective correction of the progressive exacerbation of chronic cerebral ischemia (CCI) [4]. We know that the proliferation of endothelial cells (ECs) is crucial to angiogenesis [5]. Therefore, any molecular regulation method that could be used in combination with indirect revascularization surgery to promote EC proliferation and angiogenesis should improve the surgical outcomes and prognoses of moyamoya patients. However, the molecular mechanism regulating EC proliferation under chronic ischemic conditions remains unclear.

MicroRNAs (miRNAs), which are highly conserved, single-stranded 18- to 25-nucleotide-long noncoding RNAs, can regulate target gene expression by degrading messenger RNAs and inhibiting the translation of proteins. The 5' and 3' arms of some miRNA precursors produce functionally mature miRNAs that target different sites and exhibit different biological functions; the names of the associated miRNAs are generally appended with "-5p" and "-3p", respectively [6]. Previous studies have confirmed that miRNAs are critically involved in various essential biological processes, including proliferation, development, differentiation, and apoptosis [7].

A growing number of studies have confirmed that miRNA-126 (miR-126) is the only EC-specific miRNA. miRNA-126 acts as an angiogenetic signaling regulator that plays an important role in promoting EC proliferation and migration, regulating angiogenesis, and maintaining the integrity of ECs and blood vessels [8, 9]. Because the expression of miRNA-126-3p (miR-126-3p) is higher than that of miRNA-126-5p (miR-126-5p), earlier studies have focused much more on miR-126-3p than on miR-126-5p [10–13]. However, in 2014, Schober first demonstrated that the regenerative proliferation of ECs is induced by miR-126-5p [14]. In 2017, Esser showed that the inhibition of miR-126-5p expression significantly reduces the proliferation of vascular ECs in vivo and in vitro [15]. This finding suggests that miR-126-5p might play an important role in regulating the proliferation of ECs and that increasing the expression of miR-126-5p in or around ischemic brain tissue might be an effective regulatory approach for promoting EC proliferation and angiogenesis. Therefore, we sought to construct a microenvironment of EC proliferation in a two-vessel occlusion (2VO) rat model of CCI in combination with EMS to study the regulatory effect of miR-126-5p on the proliferation of ECs in chronically ischemic brains. We transfected TM tissue with an agomir/antagomir while performing EMS and explored the possible molecular pathways acting downstream of miR-126-5p. The findings obtained in this study are expected to provide new therapeutic targets and theoretical support for improving the clinical effects of indirect revascularization surgery and the prognoses of moyamoya patients.

## RESULTS

### miR-126-5p is upregulated in TM and DM tissue of moyamoya patients, and this upregulation is associated with higher angiogenesis potential and better revascularization effects

**Figure 1:**
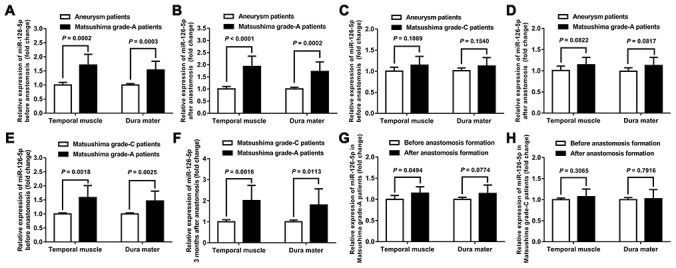
**Expression of miR-126-5p in clinical samples.** (**A**) Column chart showing the differences in miR-126-5p expression in the TM and DM samples between aneurysm patients (n = 8) and Matsushima grade-A patients (n = 8) before anastomosis formation. (**B**) Column chart showing the differences in miR-126-5p expression between aneurysm patients and Matsushima grade-A patients after anastomosis formation. (**C**) Column chart showing the differences in miR-126-5p expression between aneurysm patients and Matsushima grade-C patients (n = 8) before anastomosis formation. (**D**) Column chart showing the differences in miR-126-5p expression between aneurysm patients and Matsushima grade-C patients after anastomosis formation. (**E**) Column chart showing the differences in miR-126-5p expression between Matsushima grade-A patients and Matsushima grade-C patients before anastomosis formation. (**F**) Column chart showing the differences in miR-126-5p expression between Matsushima grade-A patients and Matsushima grade-C patients 3 months after anastomosis formation. (**G**) Column chart showing the differences in miR-126-5p expression in the TM and DM samples from Matsushima grade-A patients (n = 8) before and after anastomosis formation. (**H**) Column chart showing the differences in miR-126-5p expression in the TM and DM samples from Matsushima grade-C patients (n = 8) before and after anastomosis formation. The error bars represent the ±SDs. TM: temporal muscle; DM: dura mater.

The results showed that the expression of miR-126-5p in TM and DM tissues was significantly higher in moyamoya patients with Matsushima grade-A revascularization than in both aneurysm patients (the control group) and the patients with Matsushima grade-C anastomosis, and this finding was obtained both before and after anastomosis formation. In addition, for the Matsushima grade-A patients, the expression of miR-126-5p in contralateral TM tissue harvested 3 months after the first revascularization surgery (after anastomosis formation) was also significantly higher than that observed in TM tissue harvested during the first surgery (1.145 ± 0.154 vs. 1.008 ± 0.094, *P* = 0.0494) (Figure 1).

### miRNA-126-5p promotes HUVEC proliferation, tube formation and migration through the PI3K/Akt signaling pathway

**Figure 2:**
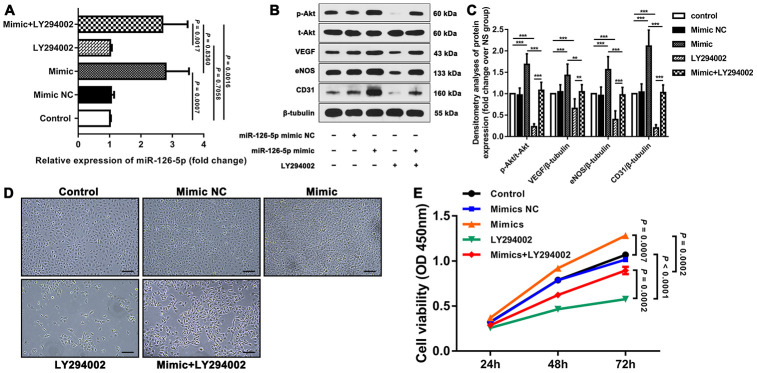
**Effect of miR-126-5p on HUVEC proliferation and downstream signaling pathways.** (**A**) qRT-PCR results showing the expression of miR-126-5p in the different groups. (**B**) Representative western blot showing the expression of p-Akt, VEGF, eNOS and CD31 in each group (normalized to the expression of β-tubulin). (**C**) Densitometry analyses of p-Akt, VEGF, eNOS and CD31 expression in each group normalized to the expression of t-Akt and β-tubulin. The error bars represent the ±SDs. *P < 0.05, **P < 0.01, ***P < 0.001. (**D**) Representative fields showing the degrees of HUVEC proliferation in each group (100× magnification). (**E**) Line graph showing the results of the cell viability CCK-8 assay. The error bars represent the ±SDs. VEGF: vascular endothelial growth factor; eNOS: endothelial nitric oxide synthase; NC: negative control.

To detect the effects of miRNA-126-5p on EC proliferation and angiogenesis and its possible downstream signaling pathway, we first examined the expression of miRNA-126-5p, p-Akt, VEGF, eNOS and CD31 in different groups of human umbilical vein endothelial cells (HUVECs) and detected the viability of the cells in these groups. The results showed that miRNA-126-5p expression was significantly higher in the mimic group than in the control group (*P* = 0.0007). The expression of miRNA-126-5p in the mimic+LY294002 group was significantly higher than those in the LY294002 group (*P* = 0.0017) and the control group (*P* = 0.0016) but did not differ from that in the mimic group (*P* = 0.8360). In addition, the expression of miRNA-126-5p in the LY294002 group was not significantly different from that in the control group (*P* = 0.7058). Western blot assays revealed that the expression levels of p-Akt, VEGF, eNOS and CD31 were significantly higher in the mimic group than in both the control and mimic+LY294002 groups. The expression of the aforementioned cytokines was significantly lower in the LY294002 group than in both the control and mimic+LY294002 groups (Figure 2A–2C). As demonstrated by CCK-8 assays, the viability of the cells in the mimic group was significantly higher than those of the cells in the control group (*P* = 0.0007) and the cells in the mimic+LY294002 group (*P* = 0.0002), and significantly lower cell viability was detected in the LY294002 group than in the control group (*P* < 0.0001) and the mimic+LY294002 group (*P* = 0.0002) (Figures 2D, 2E).

**Figure 3:**
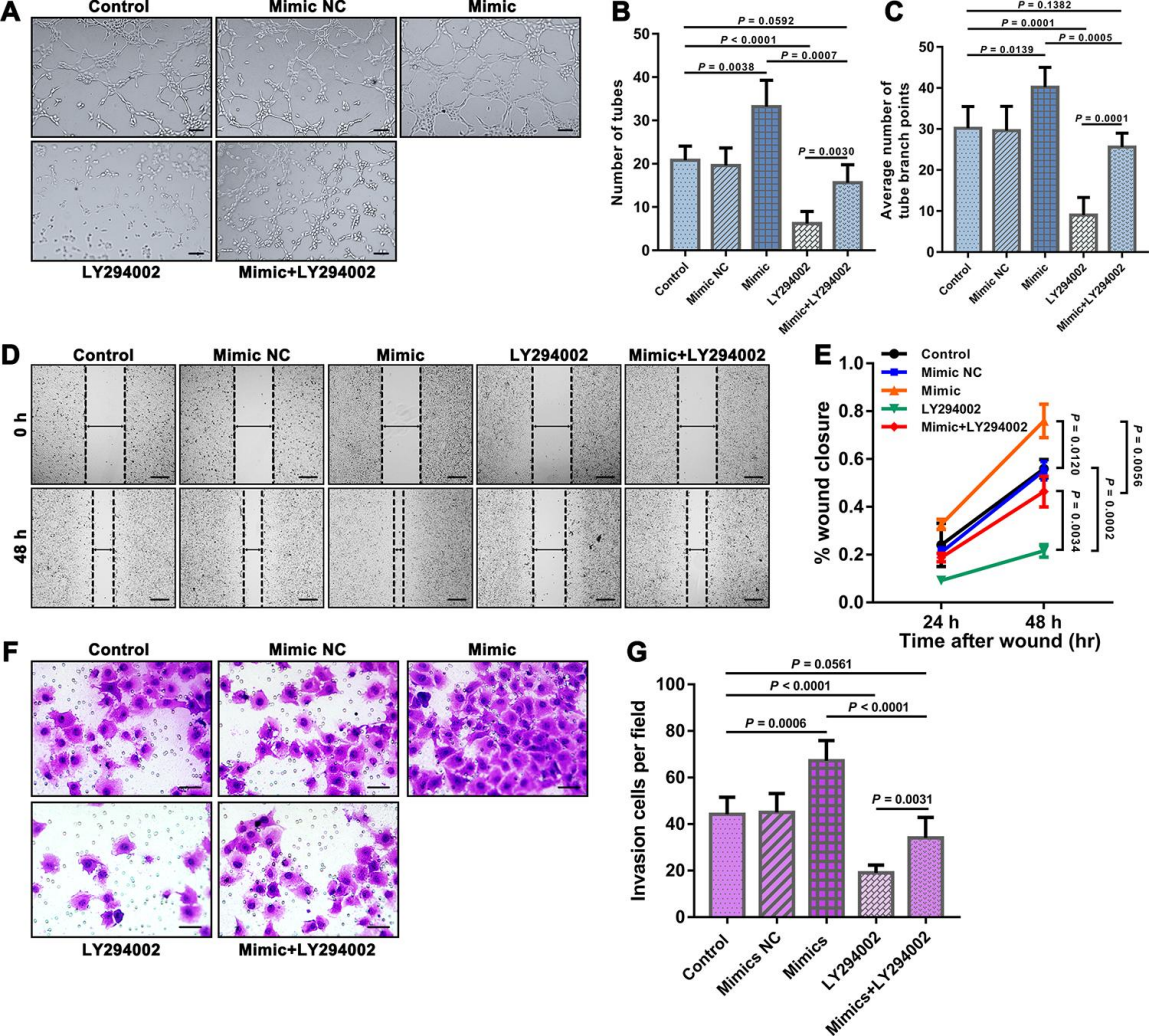
**Results of tube formation, scratch wound, and Transwell migration assays reflecting the functions of HUVEC.** (**A**) Representative fields showing the results from the tube formation assays of each group. Bar = 100 μm. The results were quantified by (**B**) the number of tubes and (**C**) the average number of tube branch points. (**D**) Representative fields showing the results from the scratch wound assays at 48 h obtained for each group. Bar = 200 μm. (**E**) The results were quantified by the percentage of wound closure at 24 and 48 h. (**F**) Representative fields showing the results from the Transwell migration assays for each group. Bar = 50 μm. (**G**) The results were quantified by the invaded cells per field. The error bars represent the ± SDs.

We further examined the tube formation and migration of HUVECs in the different groups through tube formation, Transwell migration and scratch wound assays. The results showed that the mimic group exhibited significantly improved HUVEC tube formation and migration than both the control group and the mimic+LY294002 group, and significantly worse HUVEC tube formation and migration were observed in the LY294002 group than in both the control group and the mimic+LY294002 group (Figure 3).

### EMS can induce a microenvironment of EC proliferation and improve CBP in the ischemic brains of 2VO rats

**Figure 4:**
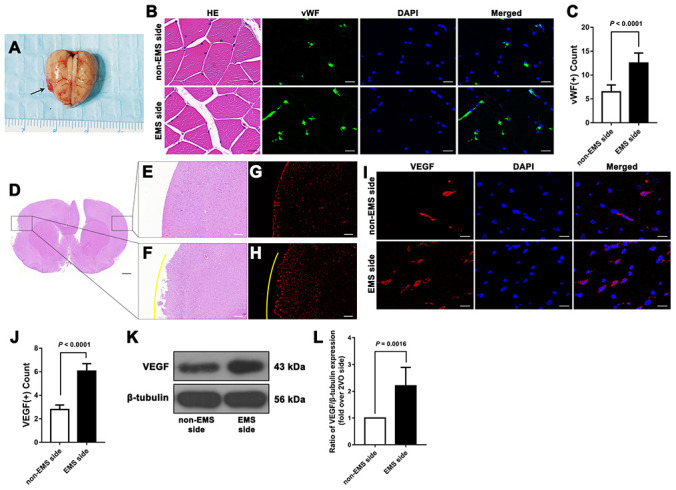
**Effectiveness of the 2VO+EMS model in promoting EC proliferation determined by immunofluorescence and western blot assays.** (**A**) Brain samples from 2VO+EMS rats (the black arrow indicates the adhered TM tissue). (**B**, **C**) Hematoxylin and eosin (HE) and immunofluorescence results showing that there was a significantly higher number of vWF(+) cells in the TM tissue on the EMS side than on the non-EMS side. Bar = 20 μm. (**D**) HE-stained slide of a brain from a 2VO+EMS rat. The two black frames indicate the portion of the brain tissue in contact with the TM and the brain tissue on the symmetrical part of the 2VO side. Bar = 1 mm. Enlarged image of the black frame on (**E**) the non-EMS side and (**F**) the EMS side. Bar = 200 μm. VEGF(+) immunofluorescence results for (**G**) the non-EMS side and (**H**) the EMS side. Bar = 200 μm. The yellow curve indicates the brain surface involved in EMS. Immunofluorescence (**I**, **J**) and western blot (**K**, **L**) results showing that there was significantly higher VEGF expression on the EMS side in the 2VO+EMS rat brains than on the non-EMS side. Bar = 20 μm. The error bars represent the ±SDs. VEGF: vascular endothelial growth factor; EMS: encephalo-myo-synangiosis; EC: endothelial cell; vWF: von Willebrand factor.

**Figure 5:**
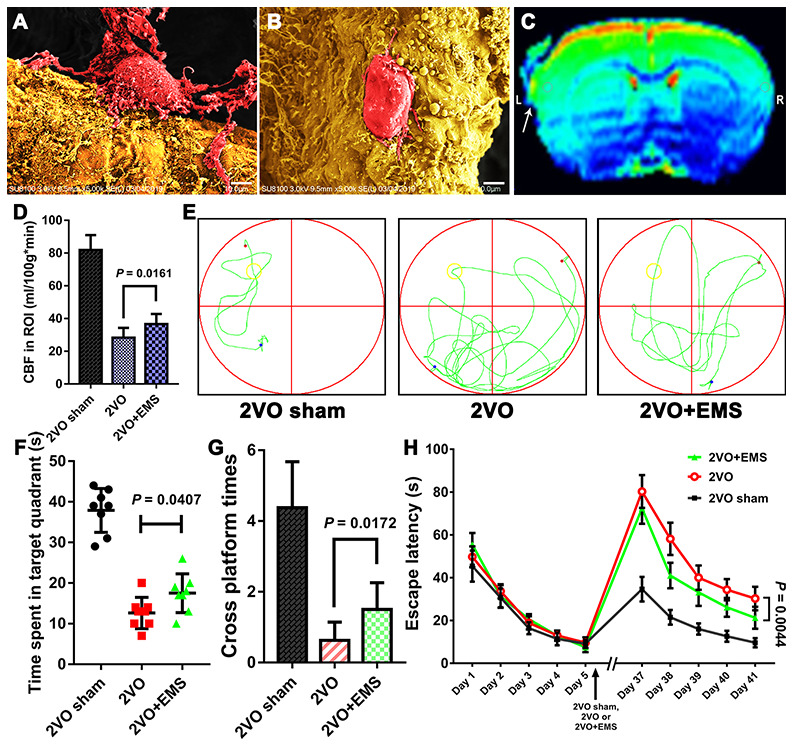
**Effectiveness of the 2VO+EMS model in improving CBP and cognitive function as determined by SEM, MRI-ASL and the MWM test.** SEM observations showed that the pericytes (**A**) in the brain cortex on the EMS side exhibited increased activity and function than those (**B**) on the non-EMS side. Bar = 10 μm. (**C**) MRI-ASL images showing a higher CBF on the EMS side than on the non-EMS side (the white arrow indicates the EMS). (**D**) Column chart showing the CBF in the 2VO sham, 2VO and 2VO+EMS groups. (**E**–**H**) The MWM test results indicated that the improvement in cognitive function observed in the 2VO+EMS group was better than that found in the 2VO group (the 2VO sham group was used as the control). The error bars represent the ±SDs. SEM: scanning electron microscopy; ASL: arterial spin labeling; MWM: Morris water maze; ASL: arterial spin labeling; EMS: encephalo-myo-synangiosis.

First, we observed that 34 days after EMS, the expression of vWF in TM on the EMS side was higher than that detected on the non-EMS side. We then compared the expression of VEGF in the brain tissues of 2VO+EMS rats (n = 8) between the EMS and non-EMS sides through immunofluorescence and western blot assays. The results showed that 34 days after EMS surgery, the expression of VEGF in the ischemic brain on the EMS side was significantly higher than that observed on the non-EMS side (6.056 ± 0.632 vs. 2.797 ± 0.379, *P* < 0.0001) (Figure 4). A scanning electron microscopy (SEM) analysis showed that the pericytes (around the capillaries) in the brain cortex on the non-EMS side exhibited atrophy with poor activity and presented finger-like extensions that were sparse and shortened, whereas the pericytes on the EMS side displayed high viability with long, abundant finger-like extensions. In addition, the CBF value of the region of interest (ROI) on the EMS side was significantly higher than that observed on the non-EMS side (36.6 ± 6.2 vs. 29.3 ± 5.1 ml/100 g·min, *P* = 0.0208). The results from the Morris water maze (MWM) test showed that the time spent in the target quadrant by the rats in the 2VO+EMS group was significantly longer than that found for the 2VO group (12.63 ± 3.85 s vs. 17.50 ± 4.74 s, *P* = 0.0407). In addition, the rats in the 2VO+EMS group crossed the platform significantly more often than the rats in the 2VO group (1.50 ± 0.76 vs. 0.63 ± 0.52, *P* = 0.0172), and the escape latency of the 2VO+EMS group was significantly shorter than that of the 2VO group (28.88 ± 5.64 vs. 30.38 ± 5.66, *P* = 0.0044) (Figure 5).

### Increasing the expression of miR-126-5p in TM tissue when performing EMS can promote the proliferation of ECs in ischemic brain tissue of 2VO rats

**Figure 6:**
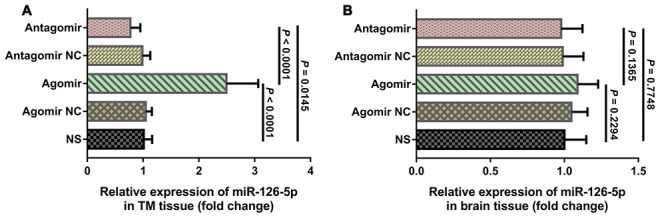
**qRT-PCR results of miR-126-5p expression in the TM and brain tissues.** (**A**) Column chart showing that miR-126-5p expression in TM tissue from rats in the agomir group was significantly higher than that observed in the NS and antagomir groups. (**B**) Column chart showing that miR-126-5p expression in brain tissue from rats in the agomir group was not significantly different from that observed in any of the other groups. The error bars represent the ±SDs. TM: temporal muscle; NS: normal saline; NC; negative control.

We used quantitative real-time polymerase chain reaction (qRT-PCR) to compare the expression of miR-126-5p in the TM and adjacent brain tissue from each group. The results showed that the expression of miR-126-5p in TM tissues from the agomir group was significantly higher than that detected in the tissues from the normal saline (NS) group (2.459 ± 0.567 vs. 1.013 ± 0.148, *P* < 0.0001), whereas the expression of miR-126-5p in TM tissues from the antagomir group was significantly lower than that detected in tissues from the NS group (0.773 ± 0.177 vs. 1.013 ± 0.148, *P* = 0.0145) (Figure 6A). However, the expression of miR-126-5p in the adjacent brain tissue from the agomir group was not significantly different from that observed in the tissues from any of the other groups (Figure 6B).

**Figure 7:**
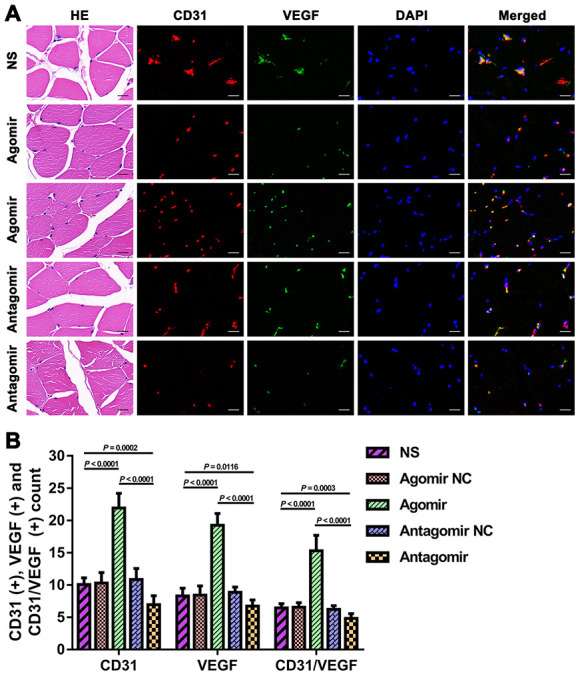
**Expression of CD31 and VEGF in TM tissues from each group.** (**A**) HE and immunofluorescence results showing CD31 and VEGF expression in TM tissues from each group. Bar = 20 μm. (**B**) Quantification of CD31(+), VEGF(+), and CD31/VEGF(+) cells in TM tissue. The data are reported as the means ± SDs. n = 8. VEGF: vascular endothelial growth factor.

**Figure 8:**
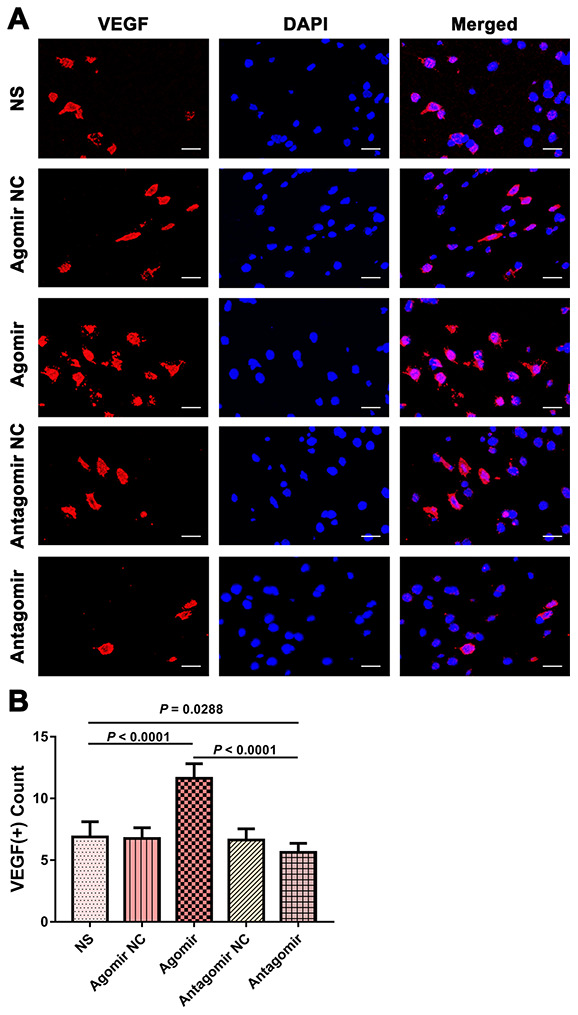
**Expression of VEGF in the brain tissues from each group.** (**A**) Immunofluorescence results showing VEGF expression in the brain tissues from each group. Bar = 20 μm. (**B**) Quantification of VEGF(+) cells in brain tissue (n = 8). VEGF: vascular endothelial growth factor.

**Figure 9:**
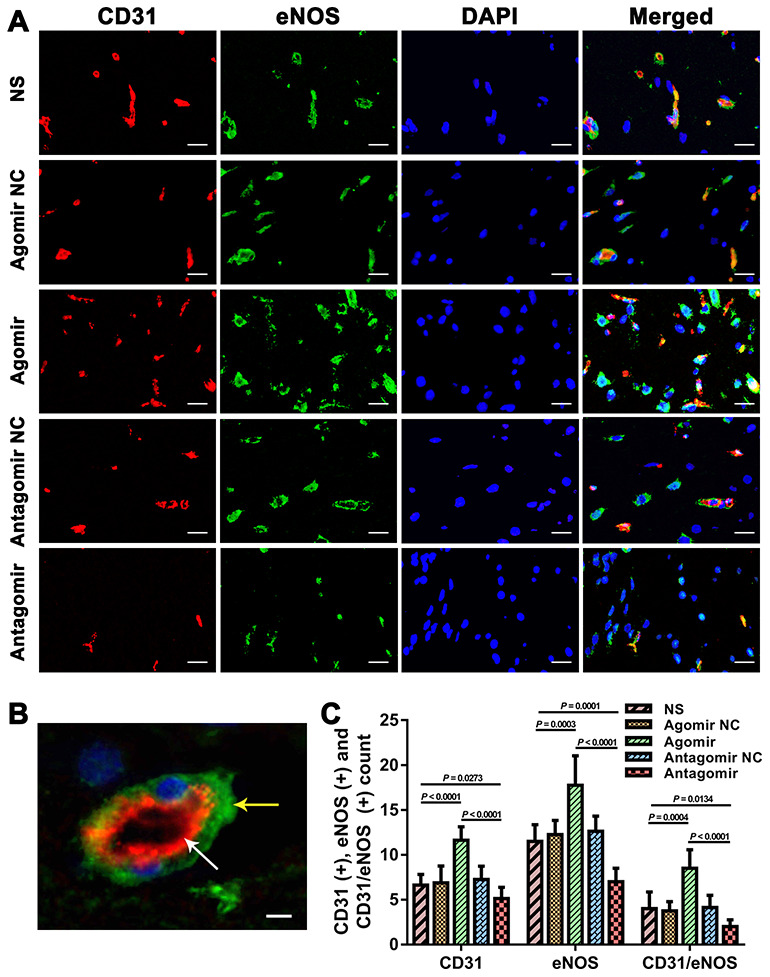
**Immunofluorescence results showing the level of CD31 and eNOS expression in the brain tissues from each group.** (**A**) Dual immunofluorescence staining showing CD31(+) and eNOS(+) cells in the ischemic brains in each group. Bar = 20 μm. (**B**) Representative dual immunofluorescence staining showing both CD31(+) and eNOS(+) cells. Bar = 5 μm. The white arrow indicates a CD31(+) cell, and the yellow arrow indicates an eNOS(+) cell. (**C**) Counts of CD31(+), eNOS(+) and CD31/eNOS(+) cells in each group. The error bars represent the ±SDs. eNOS: endothelial nitric oxide synthase.

**Figure 10:**
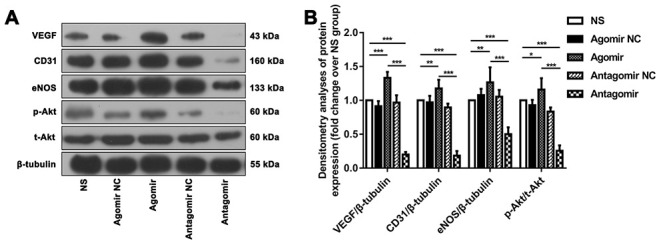
**Western blot results showing the expression of relevant proteins in each group.** (**A**) Representative western blot showing the expression of VEGF, CD31, eNOS and p-Akt in the ischemic brain tissue adjacent to the TM in each group (normalized to β-tubulin expression). (**B**) Densitometry analyses of VEGF, CD31, eNOS and p-Akt expression normalized to the expression of β-tubulin and t-Akt. The error bars represent the ±SDs. *P < 0.05, **P < 0.01, ***P < 0.001. VEGF: vascular endothelial growth factor; TM: temporal muscle; eNOS: endothelial nitric oxide synthase.

We subsequently performed immunofluorescence and western blot assays to compare the expression of VEGF, CD31, eNOS and p-Akt. The results showed that the expression of VEGF and CD31 in TM tissues from the agomir group was significantly higher than that detected in TM tissues from the NS and antagomir groups (Figure 7). Furthermore, the expression of VEGF, CD31, eNOS and p-Akt in the cerebral cortex of rats belonging to the agomir group was significantly higher than that detected in the cerebral cortex of rats belonging to the NS and antagomir groups; in contrast, the expression of VEGF, CD31, eNOS and p-Akt was significantly lower in the antagomir group than in all the other control groups (Figures 8–10).

**Figure 11:**
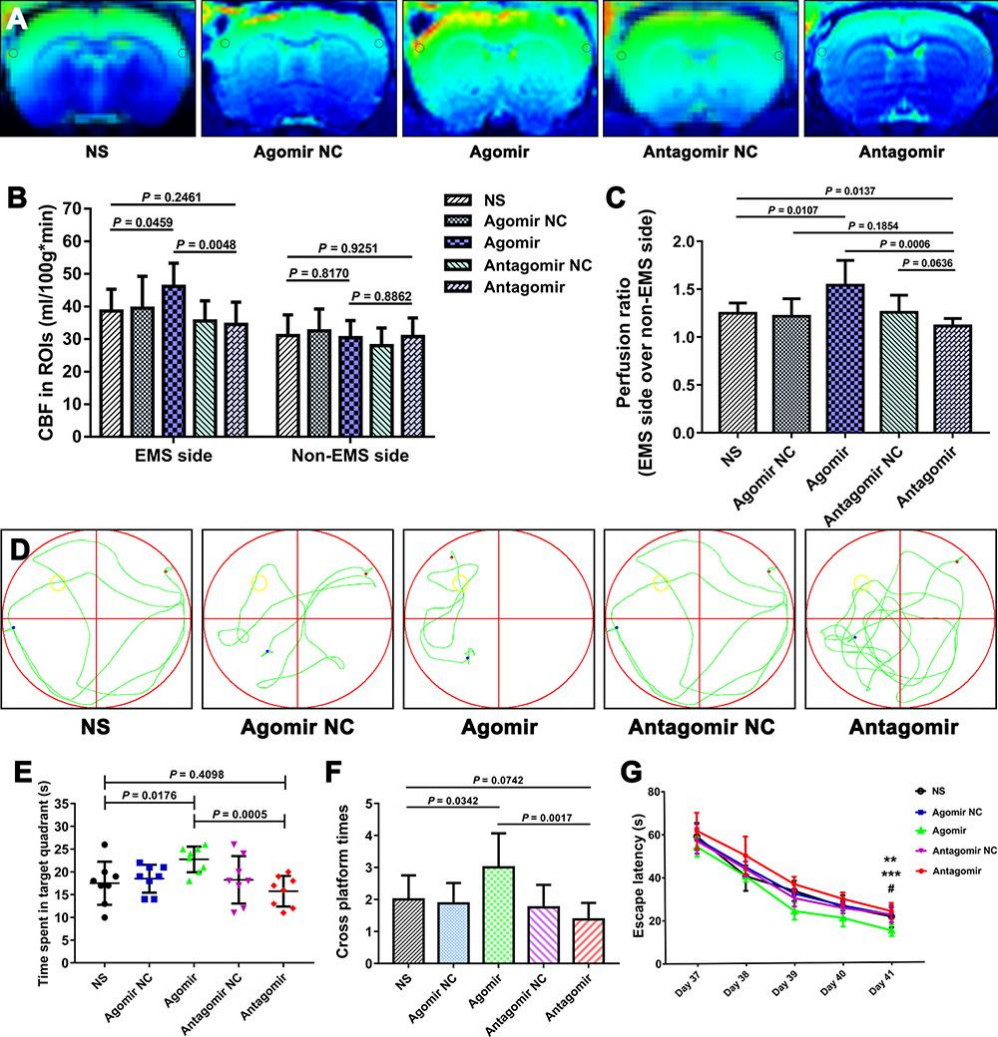
**Results of the MIR-ASL and the MWM test for each group.** (**A**) MIR-ASL films showing the CBP differences between the various group. (**B**) Column chart showing the CBP values of the ROIs (gray circles) on both sides obtained for each group. (**C**) Chart of the perfusion ratios of each group. (**D**) Representative swimming path obtained for each group. (**E**) Time spent in the target quadrant by the rats in each group. (**F**) Times crossing the platform obtained for each group. (**G**) Average escape latency obtained for each group. The error bars represent the ±SDs. ** P < 0.01 for the agomir group vs. the NS group; *** P < 0.001 for the agomir group vs. the antagomir group; # P > 0.05 for the antagomir group vs. the NS group. CBP: cerebral blood perfusion, ASL: arterial spin labeling; MWM: Morris water maze; ROI: region of interest.

We used the CBP values and perfusion ratios in the magnetic resonance imaging arterial spin labeling (MRI-ASL) sequences to compare the improvement in CBP among the various groups. The CBP values of the ROI on the EMS side obtained for the agomir group were significantly higher than those found for the NS and antagomir groups (46.15 ± 7.12 vs. 38.58 ± 6.70 ml/100 g·min, *P* = 0.0459; 46.15 ± 7.12 vs. 34.48 ± 6.84 ml/100 g·min, *P* = 0.0048). However, the CBP values of the ROIs on the EMS side obtained for the antagomir group were not significantly lower than those obtained for the NS group (34.48 ± 6.84 vs. 38.58 ± 6.70 ml/100 g·min, *P* = 0.2461). In addition, the perfusion ratio of the agomir group was 1.541 ± 0.260, which was significantly higher than that of the NS (1.248 ± 0.107, *P* = 0.0107) and antagomir (1.116 ± 0.079, *P* = 0.0006) groups. Furthermore, the perfusion ratio of the antagomir group was significantly lower than that of the NS group (1.116 ± 0.079 vs. 1.248 ± 0.107, *P* = 0.0137) (Figure 11A–11C).

Furthermore, we evaluated the improvements in cognitive function in the rats belonging to the various groups using the MWM test. The results showed that the time spent in the target quadrant by the rats belonging to the agomir group was significantly longer than that obtained for the NS and antagomir groups (22.8 ± 2.8 s vs. 17.5 ± 4.8 s, *P* = 0.0176; 22.8 ± 2.8 s vs. 15.8 ± 3.4 s, *P* = 0.0005), whereas no significant difference was observed between the antagomir and NS groups (15.8 ± 3.4 s vs. 17.5 ± 4.8 s, *P* = 0.4098). In addition, the rats belonging to the agomir group crossed the platform significantly more often than those in the NS and antagomir groups (3.0 ± 1.1 vs. 2.0 ± 0.8, *P* = 0.0342; 3.0 ± 1.1 vs. 1.4 ± 0.5, *P* = 0.0017), whereas no difference was found between the antagomir and NS groups (1.4 ± 0.5 vs. 2.0 ± 0.8, *P* = 0.0742). In addition, the escape latency on day 41 obtained for the agomir group was significantly shorter than that found for the NS and antagomir groups (14.8 ± 2.6 s vs. 21.3 ± 5.1 s, *P* = 0.0062; 14.8 ± 2.6 s vs. 23.8 ± 3.8 s, *P* < 0.0001). In contrast, the escape latency of the antagomir group was not significantly different from that obtained for the NS group (23.8 ± 3.8 s vs. 21.3 ± 5.1 s, *P* = 0.2848) (Figures 11D–11G).

## DISCUSSION

The present study provides the first demonstration that increasing the expression of miR-126-5p in TM tissue can promote EC proliferation and angiogenesis in chronically ischemic brain tissue of 2VO+EMS rats, improve CBP in ischemic brains and facilitate the recovery of cognitive function. In addition, this study confirms the feasibility of the 2VO+EMS rat model for inducing a microenvironment of angiogenesis in chronically ischemic brain tissue and for establishing an approach for the delivery of gene products and extending their effects from the TM to adjacent brain tissue. Finally, this study reveals that miR-126-5p promotes angiogenesis via the PI3K/Akt pathway.

### miR-126-5p is effective in promoting EC proliferation and angiogenesis in chronically ischemic brain tissue

miR-126, which is specifically expressed in vascular ECs, regulates EC proliferation, migration and angiogenesis [8, 9]. The 5' and 3' arms of the miR-126 precursor produce functionally mature miRNAs named miR-126-3p and miR-126-5p, respectively. Because the expression of miR-126-3p is higher than that of miR-126-5p, researchers were initially more interested in miR-126-3p [16]. For example, some studies have shown that miR-126-3p directly inhibits the target genes SPRED1, VCAM1 and PIK3R2 to promote EC proliferation and angiogenesis [10–13]. However, Schober’s research group demonstrated that 1) the decrease in EC proliferation in miR-126-knockout mice is due to a lack of miR-126-5p but not miR-126-3p and 2) the regenerative proliferation of ECs is induced by miR-126-5p alone [14]. Esser’s team subsequently showed that the inhibition of miR-126-5p expression significantly reduces the proliferation of vascular ECs in vivo and in vitro [15]. Thus, compared with miR-126-3p, miR-126-5p mighty be a mature miR-126 that truly promotes the proliferation of ECs.

After assessing the expression of miR-126-5p in the TM and DM (blood-supplying tissue) of moyamoya patients, we observed that moyamoya patients that exhibit better recanalization effects present higher miR-126-5p expression in their TM and DM tissues both before and after the formation of EC-IC anastomosis compared with those that experience worse recanalization effects. Furthermore, in moyamoya patients with better recanalization effects, the expression of miR-126-5p in the TM tissue might be higher after anastomosis formation. These findings suggest that miR-126-5p might play a role in promoting EC proliferation and angiogenesis in chronically ischemic brain tissue. In addition, we explored the function of miRNA-126-5p in angiogenesis using in vitro models and found that the overexpression of miRNA-126-5p promoted the proliferation, tube formation, and migration of HUVECs through the PI3K/Akt signaling pathway. Furthermore, we performed EMS surgery on 2VO rat models and transfected their TM tissue with miR-126-5p agomir/antagomir. The results showed that the expression of miR-126-5p in TM tissue was significantly higher in the agomir group than in any of the other groups. Accordingly, the expression levels of EC proliferation-related cytokines (VEGF), EC markers (CD31) and EC repair-related cytokines (eNOS) in adjacent ischemic brain tissue from the agomir group were significantly higher than those observed in the control group. Furthermore, the greatest improvements in CBP and cognitive function were detected in the agomir group. In contrast, the levels of VEGF, CD31 and eNOS expression in ischemic brain tissue from the antagomir group were lower than those observed in the other groups, and MRI-ASL exhibited that this group presented the lowest improvement in CBP. The above-mentioned results suggest that increased miR-126-5p expression in the TM can promote the proliferation of ECs and the process of angiogenesis in adjacent ischemic brain tissues (blood-receiving tissues) after EMS surgery. In addition, we observed that this biological effect of miR-126-5p further improved the CBP values in chronically ischemic brains and thereby promoted the recovery of cognitive function. This finding provides new ideas for improving the surgical outcomes of patients with chronic ischemic cerebral disease such as moyamoya disease. However, we also noted that the recovery of cognitive function was not significantly worse in the antagomir group than in the other control groups. This outcome was probably obtained because the physical exercise exerted by rats during the MWM test can also promote EC proliferation, and this effect might have neutralized the negative effects of the miR-126-5p antagomir.

### Transfection of the TMs of 2VO+EMS rats with agomir is a novel strategy for the delivery of gene products and extending their effects to chronically ischemic brains

Establishing a microenvironment of EC proliferation in ischemic rat brain tissue can aid exploratory research on the mechanisms associated with recovery after ischemic stroke. Therefore, we performed EMS surgery on a 2VO rat model to simulate indirect revascularization surgery for the clinical treatment of moyamoya disease. The results showed that the expression of VEGF in brain tissue on the EMS side was significantly higher than that on the contralateral side, and the MRI-ASL sequence also suggested that the CBP on the EMS side was better than that on the contralateral side. These findings are sufficient to support the feasibility of the 2VO+EMS method for establishing an EC proliferation microenvironment in chronically ischemic brain tissue.

Given the successful establishment of 2VO+EMS animal models, we can further explore the effects of experimental interventions on EC proliferation and angiogenesis in ischemic brain tissue. Interventions that act directly on brain tissue (such as direct intracerebral injection), even if proven effective, cannot be applied directly in clinical settings due to medical ethics considerations. However, due to the advantage of EMS surgery, we can directly apply these interventions to TM tissue and then wait for the effects of the intervention to gradually extend into the brain tissue along the anastomotic vessels from the TM to the brain. This approach not only avoids direct damage to the brain but also exerts a therapeutic effect on adjacent ischemic brain tissue; thus, this approach could be used in the clinic.

Based on the 2VO+EMS model and the “TM-to-brain” intervention approach, we transfected the TM tissue of 2VO+EMS rats with miR-126-5p agomir/antagomir, and the results showed that increasing the expression of miR-126-5p in the TM can upregulate the proliferation of ECs in chronically ischemic brain tissue, which is closely covered by the TM. Therefore, this finding suggests that transfection of the TM of 2VO+EMS rats with agomir is a novel strategy for the delivery of gene products and extending their effects into chronically ischemic brain tissue. In addition, this method could be used in the future to deliver genetic products, such as angiogenic growth factors, to the TM or muscle/brain interface with the aim of improving revascularization after EMS surgery in moyamoya patients.

### Downstream molecular mechanism through which miRNA-126-5P promotes EC proliferation

In this study, we confirmed the effect of miRNA-126-5p on EC proliferation and downstream signaling pathways through in vitro experiments. First, we found that miR-126-5p overexpression could significantly promote HUVEC proliferation and increase p-Akt expression. However, culturing HUVECs with LY294002 (PI3-kinase inhibitor) significantly lowered p-Akt expression and inhibited HUVEC proliferation but hardly had an impact on miR-126-5p expression. This result indicated that the PI3K/Akt pathway is downstream of miR-126-5p and positively regulates EC proliferation. In addition, miR-126-5p overexpression significantly increased the expression of p-Akt, VEGF, CD31 and eNOS. However, LY294002, which is an inhibitor of the PI3K/Akt pathway, could also lower the expression of VEGF, eNOS and CD31. This result indicated that VEGF, CD31 and eNOS play a role in promoting EC proliferation downstream of the PI3K/Akt pathway.

In the animal experiments, the agomir group was superior to the other groups in terms of both CBP improvement and cognitive function recovery. We believe that this finding was obtained due to the promotion of EC proliferation and angiogenesis by miR-126-5p. Through immunofluorescence and western blot assays, we found that the high degree of CBP improvement on the EMS side observed in the agomir group was consistent with high expression of p-Akt and its factors downstream VEGF, CD31 and eNOS, which indicates that the PI3K/Akt signaling pathway might play a positive role in promoting the expression of VEGF, CD31 and eNOS downstream of miR-126-5p in chronically ischemic brains [16, 17].

**Figure 12:**
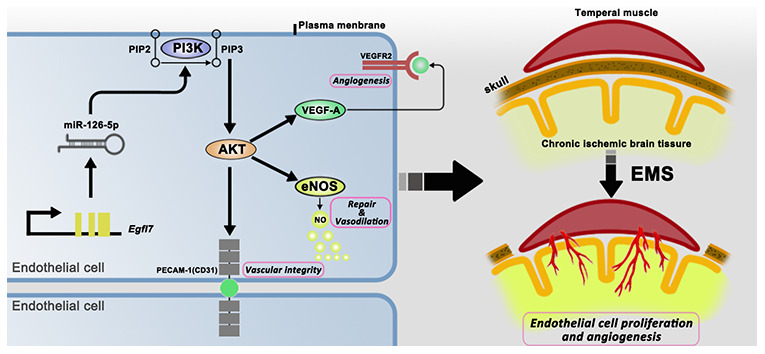
**Schematic showing the signaling pathways that act downstream of miR-126-5p to promote EC proliferation and angiogenesis.**

VEGF is a well-known angiogenesis-promoting cytokine that plays important roles in the proliferation and migration of ECs and in angiogenesis [18–20]. CD31 (platelet endothelial cell adhesion molecule-1, PECAM-1), which is an EC marker located at tight junctions between ECs, is used in most cases to prove the existence of ECs and angiogenesis [19–23]. The high expression of these molecules in the miR-126-5p-overexpression groups detected both in vitro and in vivo suggests that miR-126-5p promotes EC proliferation by increasing the expression of downstream VEGF and CD31. eNOS produces nitric oxide in blood vessels to help regulate vascular function and participates in the regulation of EC repair and proliferation [24–28]. The highest levels of eNOS were observed in the miR-126-5p-overexpression groups both in vitro and in vivo, which suggested that miR-126-5p can increase the secretion of downstream eNOS and promote the repair of ECs damaged by chronic ischemia (Figure 12).

### Limitations

There may be several limitations. First, the target gene(s) through which miRNA-126-5p promotes EC proliferation was(were) not clarified. Second, our study could not clarify whether some of the proliferation of ECs was attributable to physical exercise during the MWM test. In addition, the mechanism through which the effect of miR-126-5p in promoting EC proliferation spread from the TM to the ischemic cortex is unclear. Finally, the most effective transfection dose for miRNA-126-5p agomir injection remains unexplored.

## CONCLUSION

After establishing a 2VO+EMS rat model, we showed that increasing the expression of miR-126-5p in TM tissue can promote EC proliferation and angiogenesis in adjacent chronically ischemic brain tissue. This process not only improves regional CBP in rats but also promotes the recovery of cognitive function. The role of miRNA-126-5p in promoting EC proliferation is attributed to the increase in the expression of VEGF, CD31 and eNOS mediated through the PI3K/Akt pathway.

## MATERIALS AND METHODS

### TM and DM tissue collection and postoperative assessments

**Figure 13:**
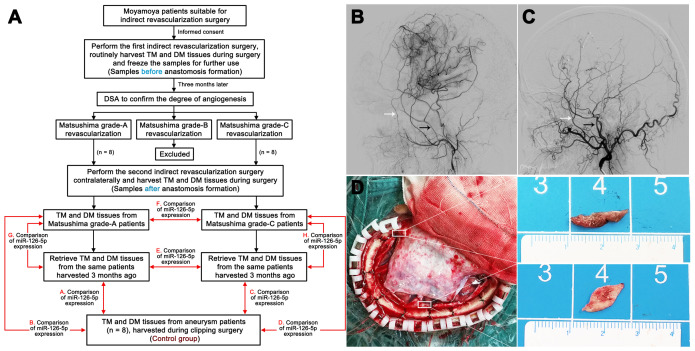
**Harvesting of clinical samples.** (**A**) Schedule of TM and DM sample harvesting. DSA films showing Matsushima grade-A revascularization (**B**) and Matsushima grade-C revascularization (**C**). The white arrows indicate the deep temporal artery, and the black arrows indicate the middle meningeal artery. (**D**) Intraoperative image showing the sources of the TM and DM samples. TM: temporal muscle; DM: dura mater.

We initially explored the relationship between the expression of miR-126-5p and the degree of angiogenesis after indirect revascularization surgery by detecting the expression of miR-126-5p in TM and DM tissues from moyamoya patients (Table 1). During the combined EMS and dura inversion procedure for moyamoya patients, samples of the TM (1.0 × 0.5 cm) and DM (1.0 × 1.0 cm) were collected and stored routinely for further use (pre-anastomosis formation samples). All fresh samples were used for qRT-PCR, frozen in liquid nitrogen and stored at -80°C. Three months after the initial revascularization surgery, we routinely performed digital subtraction angiography (DSA) examinations to detect the formation of extracranial-to-intracranial (EC-IC) anastomosis based on Matsushima classification scores: grade-A revascularization indicated that more than two-thirds of the middle cerebral artery (MCA) anastomosis-induced circulation was fulfilled, grade-B revascularization indicated one-third to two-thirds fulfillment, and grade-C revascularization indicated less than one-third fulfillment [29]. We then harvested TM and DM samples from patients with grade-A revascularization (n = 8) and grade-C revascularization (n = 8) during contralateral indirect revascularization surgery (post-anastomosis formation samples) and compared their miR-126-5p expression levels (Figure 13). The TM and DM samples were also harvested from the aneurysm patients (n = 8) during clipping procedurals, and the miR-126-5p expression of these samples was used as the control. The study was carried out in accordance with the Declaration of Helsinki (2000) and was approved by the Ethics Committee of the Third Affiliated Hospital of Sun Yat-sen University. Written informed consent was obtained from all the participants.

**Table 1:** Baseline data of the included moyamoya and aneurysm patients.

	**Aneurysm patients (n = 8)**	**Matsushima grade-A patients (n = 8)**	**Matsushima grade-C patients (n = 8)**	***P* value**
Age (years)	54.8 ± 9.3	49.8 ± 12.3	45.3 ± 12.5	0.2756
Sex (male:female)	4:4	3:5	2:6	0.5866
Hypertension (%)	50.0 (4/8)	50.0 (4/8)	75.0 (6/8)	0.5037
T2DM (%)	50.0 (4/8)	12.5 (1/8)	25.0 (2/8)	0.2437
Smoking (%)	25.0 (2/8)	25.0 (2/8)	12.5 (1/8)	0.7768
Past infarctions (%)	25.0 (2/8)	12.5 (1/8)	12.5 (1/8)	0.7408
Past TIAs (%)	50.0 (4/8)	67.5 (5/8)	37.5 (3/8)	0.6065
Past hemorrhages (%)	0(0/8)	12.5 (1/8)	25.0 (2/8)	0.3189
Past seizures	12.5 (1/8)	12.5 (1/8)	25.0 (2/8)	0.7408
Suzuki grade (left)				0.8559
2	/	4	5	
3	/	3	2	
4	/	1	1	
Suzuki grade (right)				0.6422
1	/	1	0	
2	/	4	3	
3	/	2	3	
4	/	1	2	
mRS before surgery				0.8614
0	1	2	2	
1	6	4	5	
2	1	2	1	

T2DM: type 2 diabetes mellitus; TIA: transient ischemic attack; mRS: modified Rankin Scale. The age data are expressed as the means ± SDs. The Suzuki grades were evaluated according to the criteria described by Suzuki and Kodama (PMID: 6823678).

### Cell culture, transfection and subgroup

HUVECs were obtained from Shanghai Institute of Life Sciences, Chinese Academy of Sciences, and were routinely cultured in M200 medium containing 2% FBS at 37°C in an atmosphere with 5% CO^2^/95% air. All the experiments were performed using HUVECs between passages 3 and 8. Once the cells reached 70–80% confluency, miR-126-5p mimics (RiboBio, Guangzhou, China) or the negative control for the miR-126-5p mimics (mimics NC; RiboBio, Guangzhou, China) were transfected into HUVECs using Lipofectamine® 2000 (Invitrogen; Thermo Fisher Scientific Inc.) according to the manufacturer’s recommended protocols. The HUVECs were divided into the following five groups: 1) control group, 2) mimic NC group, 3) mimic group, 4) LY294002 group, and 5) mimic+LY294002 group (Table 2). The transfection efficiency and miR-126-5p expression in each group were detected by qRT-PCR (in detail below), and the expression of p-Akt, VEGF, eNOS, and CD31 in each group was examined by western blot (in detail below).

**Table 2:** HUVEC groups.

**Groups**	**Descriptions**
Control	Naïve
Mimic NC	Transfected with 50 nM miRNA-126-5p mimic negative control
Mimic	Transfected with 50 nM miRNA-126-5p mimic
LY294002	Treated with 10 μM LY294002
Mimic+LY294002	Transfected with 50 nM miRNA-126-5p mimic and treated with 10 μM LY294002

### Cell viability assay

The HUVEC viability was determined by the CCK-8 assay (Dojindo, Kumamoto, Japan). Briefly, the cells were seeded into 96-well plates at a density of 3 × 10^3^ cells per well and grown overnight. After the indicated treatments, the HUVECs were washed with phosphate-buffered saline (PBS), and 10 μL of CCK-8 solution was added to each well. The plates were then incubated for 2 h at room temperature and then for 24, 48 and 72 h. The optical density (OD) values at w wavelength of 450 nm were then determined to measure the HUVEC viability. Each experiment was performed in triplicate and repeated at least three times.

### Tube formation assay

The ability of HUVECs to form capillary-like tubes in culture was assessed by adding 4 × 10^5^ cells to each well of a 48-well plate, and each well in the plate contained 200 μL of pre-gelled Matrigel (BD Biosciences, San Jose, CA, USA) in 1 mL of complete medium. The HUVECs were transfected with miR-126-5p mimics or treated with LY294002. The plates were then incubated at 37°C for 6 h to form capillary-like structures. The degree of tube formation was quantified by measuring the number of tubes and number of branch points in five randomly selected fields from each well (×100). Each experiment was performed at least three times.

### Scratch wound assay

The effects of miR-126-5p mimics on the migration of HUVECs were evaluated using the scratch wound assay. Forty-eight hours after transfection with the miR-126-5p mimic, a confluent monolayer of HUVECs (1 × 10^5^ cells/mL in a six-well plate) was wounded using a 200-μL yellow micropipette tip and washed with PBS. After incubation for 24 and 48 h, the degree of wound closure was observed under an inverted phase contrast microscope, and images were then captured in digital format (×100). The wound width was determined using Image-Pro Plus software 5.1 (Media Cybernetics, Inc. Siler Spring, MD, USA) for quantitative assessment. Each experiment was performed at least three times.

### Transwell migration assay

The effects of the miR-126-5p mimics on the migration of HUVECs were also assessed using a Transwell migration assay. Forty-eight hours after transfection with the miR-126-5p mimic, the HUVECs were suspended in serum-free medium at a concentration of 2 × 10^5^ cells/mL and placed in the upper chamber of the Transwell. The chamber was then transferred to a well containing complete medium. The membranes were allowed to migrate for 24 h and then stained with crystal violet dye. The cells in the top well were removed, and the number of remaining cells were determined by counting the cells in at least five random microscopic fields per well (×200). Three wells of cells were used for each group. Each experiment was performed at least three times.

### Animals and subgrouping

**Figure 14:**
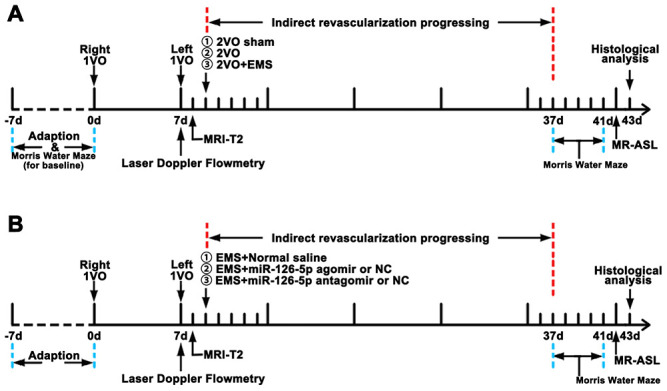
**Experimental schedule.** (**A**) Schedule used for observing the effects of EMS on EC proliferation, CBP improvement and cognitive function improvement in 2VO+EMS rats. (**B**) Schedule used for observing the effects of miR-126-5p on EC proliferation, CBP improvement and cognitive function improvement in 2VO+EMS rats. EMS: encephalo-myo-synangiosis; CBF: cerebral blood flow; EC: endothelial cell.

Adult male Sprague-Dawley rats (male, 250-300 g) were purchased from the animal center of Sun Yat-sen University, Guangzhou, China. The animals were adapted to the environment for 1 week before the experiments. All animal treatments and experiments were approved by the Institutional Animal Ethics Committee of Sun Yat-sen University, and their treatment conformed to the Guide for the Care and Use of Laboratory Animals of the National Institutes of Health (Publication No. 80-23, revised 1996). Twenty-four rats were used to detect the proliferation-promoting effects of EMS on ECs in the 2VO+EMS rat model (2VO sham group, 2VO group and 2VO+EMS group) (Figure 14A). Based on the different materials injected and transfected into the TM during EMS, the remaining rats were randomly divided into the following groups: 1) NS group, 2) agomir negative control (NC) group, 3) agomir group, 4) antagomir NC group, and 5) antagomir group (Table 3). The experimental schedule is presented in Figure 14B.

**Table 3:** Animal groups.

**Groups (n = 8)**	**Procedures**
2VO sham	Subjected to the same procedures as 2VO but without ligation of the common carotid arteries (CCAs)
2VO group	Ligation of both CCAs
2VO+EMS group	2VO plus encephalo-myo-synangiosis
NS group	2VO+EMS with the NS injected into the TM
Agomir NC group	2VO+EMS with miRNA-126-5p agomir NC injected and transfected into the TM
Agomir group	2VO+EMS with miRNA-126-5p agomir injected and transfected into the TM
Antagomir NC group	2VO+EMS with miRNA-126-5p antagomir NC injected and transfected into the TM
Antagomir group	2VO+EMS with miRNA-126-5p antagomir injected and transfected into the TM

2VO: 2-vessel occlusion; EMS: encephalo-myo-synangiosis; NS: normal saline; TM: temporal muscle; NC: negative control.

### Animal model of 2VO

**Figure 15:**
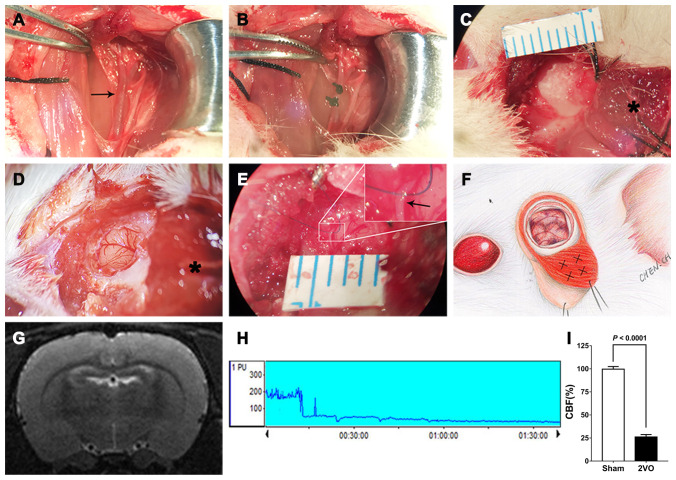
**Procedures used for establishment of the 2VO+EMS rat model.** (**A**, **B**) Procedures used for CCA ligation (the black arrow indicates the left CCA). Representative images showing the EMS procedures, including (**C**) reflection of the skin and TM from the skull, (**D**) opening of the dura (the black asterisk indicates the TM tissue) and (**E**) stitching of the TM and DM with 10-0 Prolene (the black arrow indicates the DM). (**F**) Schematic drawing showing the EMS procedure and the four injection sites for the miR-126-5p agomir in the TM. (**G**) MRI-T2 film showing a lack of infarction after the 2VO procedures. (**H**) Representative graph of CBF changes before and after 2VO. **(I)** Doppler flowmetry results showing that the CBF values in 2VO rats (n = 8) decreased to 25.8 ± 2.6% of the baseline levels obtained for the 2VO sham group (n = 8). The error bars represent the ±SDs. EMS: encephalo-myo-synangiosis; TM: temporal muscle; DM: dura mater; CBF: cerebral blood flow; CCA: common carotid artery.

A CCI model was established by performing 2VO. Each rat was anesthetized with 10% chloral hydrate, fixed on a stereotaxic instrument and placed on a heating pad, and its temperature was monitored and maintained at 37°C. The right common carotid artery (CCA) was exposed by a straight incision in the middle of the neck, and the incision was closed after ligation of the right CCA with two 3-0 silk threads. After 7 days, a scalp incision was made along the midline, a burr hole was made with an electric drill in the right frontal region (0.8 mm posterior and 3.4 mm lateral to the bregma), and a placement device fixed with cyanoacrylate adhesives for the contact probe was set. Subsequently, the left CCA was exposed using the same method. The left CCA was temporarily clamped with an artery clip for 5 min, and the clip was then released to restore the blood flow. After 5 min, the left CCA was clamped again for 10 min, and 10 min later, the left CCA was ligated with two 3-0 silk threads. The 2VO sham group was subjected to the same procedure but without common carotid artery ligation. The cerebral blood flow (CBF) before and after 2VO was measured by laser Doppler flowmetry. The mean CBF values are expressed as percentages of the baseline value. Successful 2VO model establishment was confirmed by 1) a decline in CBF to approximately 30% of the baseline before surgery and 2) lack of a large area of cerebral infarction on the brain MRI-T2 sequence 1 day after the 2VO procedure (Figure 15).

### Procedures for EMS surgery in rats

Two days after 2VO modeling, we performed EMS on the left cerebral hemispheres of 2VO rats. Each rat was anesthetized with 10% chloral hydrate, and the skin and TM were reflected in a U-shape from the skull. A piece of skull approximately 4 to 5 mm in diameter was removed from the temporoparietal region using an electric drill. The DM was carefully opened with microforceps and scissors without damaging the brain surface. Under the microscope, a 1-mL syringe needle was used to open the arachnoid in multiple places. The TM and DM were stitched together to ensure that the TM was in close contact with the ischemic brain surface, and the skin was then sutured (Figure 15). We tested the beneficial effects of EMS on EC proliferation and angiogenesis by comparing the VEGF expression, CBF measurements and pericyte activity between the different sides of the brain in 2VO+EMS rats and by comparing the cognitive function between the 2VO and 2VO+EMS groups.

### Transfection of the TM in rats

For the in vivo study, we purchased a commercially available miR-126-5p agomir, miR-126-5p antagomir, and relevant NC from RiboBio. The sequences of the miR-126-5p agomir and antagomir were as follows: miRNA-126-5p agomir, 5’-CGAGUCCCCGACCUCUCUAC-3’, and miR-126-5p antagomir, 5’-AGUUCUCAAACCCAUGGAAUUC-3’ (RiboBio). The agomir (8 μg in 8 μL of NS), antagomir (8 μg in 8 μL of NS) or NC (same amount) were mixed with 4 μL of Entranster in vivo transfection reagent (Engreen, Beijing, China) for 5 s, and the mixtures were incubated at 37°C for 15 min. Each mixture was then divided into four equal parts and injected into four sites of the TM with a Hamilton microinjector just before the TM and DM were stitched together (Figure 15F). The entire injection period lasted 30 s. The dose was based on the manufacturer’s recommendation (Engreen) and a modification of the dose reported for the local injection of agomir. The NS (same amount, same method) was injected into the TM in the 2VO+EMS NS group as a control. After the skin was sutured, the region of the TM was gently massaged for 1 min.

### Morris water maze (MWM) test

We used the MWM test to analyze the cognitive impairment caused by 2VO and the cognitive improvement after EMS and miRNA transfection [30, 31]. A circular black pool with a diameter of 180 cm and a depth of 60 cm was filled with water at 22 ± 2°C to a depth of 30 cm. The pool was divided into four equally spaced quadrants. During the training period, the center of quadrant III (QIII) hid an escape platform (10×10 cm, 1 cm below the water surface), and this platform was removed for the escape latency test. The rats were gently placed into the water and released facing the wall of one of the four quadrants, which was randomly selected. The rats were allowed to find the escape platform for 120 s, and the latency to escape onto the platform and the escape route were recorded. If a rat failed, it was guided onto the platform with a stick, and its latency time was recorded as 120 s. The training was conducted twice a day for 4 days, with intertrial intervals of approximately 20 min. The escape latency, time spent in the target quadrant and cross platform times were measured and analyzed using a video surveillance system (SMART, Panlab SL, Barcelona, Spain).

### Magnetic resonance imaging (MRI)

MRI T2 sequence measurements were obtained 24 h after 2VO to exclude visible cerebral infarction, and arterial spin labeling (ASL) sequence measurements were obtained 32 days after EMS to assess the CBP values of the rats in the different groups. The rat brains were tested using a 7.0-T MRI animal scanner (PharmaScan MRI with ParaVision 7 system, Bruker, Germany). The MRI parameters were set as follows: echo spacing = 11 ms, repetition time (TR) = 2500 ms, echo time (TE) = 33 ms, TR/TE = 2500/33 ms, field of view (FOV) = 35 × 35 mm^2^, matrix size = 256 × 256, section thickness = 0.8 mm without a gap, and scan time = 2 min 40 s. The ROIs were located within the brain cortex in close contact with the TM after EMS (and within the cortex of the symmetrical position in the contralateral hemisphere), and each ROI had an area of 10 mm^2^. The CBF value (ml/100 g·min) of each ROI was calculated using ParaVision software, and the ratio of the CBF on the EMS side to that on the non-EMS side was calculated and set as the “perfusion ratio”.

### qRT-PCR

Total RNA was extracted with the TRIzol reagent (Invitrogen, Shanghai, China) and reverse transcribed using a one-step qRT-PCR kit (TransGen Biotech Co., Ltd., China) according to the manufacturer’s instructions. cDNA was synthesized from 500 ng of RNA using a Prime Script RT Reagent Kit (TaKaRa, Otsu, Shiga, Japan). qRT-PCR was performed with a SYBR® Prime Script™ RT-PCR Kit (TaKaRa). All the primers were obtained from Invitrogen and were designed using reference sequences published by the National Center for Biotechnology Information. The sequences of the specific primers (Invitrogen, Thermo Fisher Scientific, Inc.) were as follows: miR-126-5p, 5’-CCGACACGGGAGACAATG-3’ (forward) and 5’-TCTGGAAGTGAGCCAATGTG-3’ (reverse); and U6, 5’-CTGTGCCCATCTACGAGGGCTAT-3’ (forward) and 5’-TTTGATGTCACGCACGATTTCC-3’ (reverse). The reaction conditions were 95°C for 10 min followed by 40 cycles of 95°C for 10 s, 60°C for 20 s and 72°C for 15 s. The relative expression levels were calculated using the comparative 2^-ΔΔCt^ method, and the levels of miR-126-5p were normalized to those of U6. All the experiments were performed in triplicate.

### Immunofluorescence

To determine the effects of EMS and miR-126-5p on EC proliferation, an immunofluorescence assay was performed. Briefly, sections were fixed in 4% paraformaldehyde in phosphate-buffered saline (PBS) for 5 min at room temperature, permeabilized, and blocked for 30 min with 0.1% Triton X-100 and 1% bovine serum albumin. Subsequently, the fixed sections were washed and incubated for 1 h with primary antibodies against vWF (1:200; Cell Signaling Technology, Shanghai, China), VEGF (1:200; Cell Signaling Technology), CD31 (1:100; Santa Cruz Biotechnology, Dallas, TX, USA), or eNOS (1:200; Cell Signaling Technology) overnight at 4°C. The sections were then washed three times with PBS and incubated with fluorescence-conjugated species-specific secondary antibodies. The samples were subsequently counterstained with 4’,6-diamidino-2-phenylindole (DAPI, Beyotime Biotechnology, Shanghai, China) for identification of the nuclei. Photographs were acquired using a fluorescence microscope. The quantities of vWF-, VEGF- and CD31-positive cells indicated the proliferation and distribution of capillary ECs. The quantities of eNOS-positive cells indicated the repair and proliferation of capillaries. For each group, the numbers of positively stained cells in five random visual fields (100 μm × 100 μm) were counted. All the experiments were performed in triplicate.

### Western blot analysis

We performed western blot assays to determine the expression of proteins related to EC proliferation. Total proteins were extracted with a tissue lysis kit (KeyGEN, Nanjing, China), and the protein concentrations were measured with a BCA protein assay kit (Beyotime). Subsequently, 30 μg of protein was separated by SDS-PAGE, and the separated proteins were then transferred onto a PVDF membrane (Beyotime). After blocking with Quickblock Blocking Buffer (Beyotime), the membrane was incubated with primary antibodies against VEGF (1:500; Cell Signaling Technology), CD31 (PECAM-1) (1:300; Santa Cruz Biotechnology), eNOS (1:500; Cell Signaling Technology), phospho-Akt (Ser473) (1:1000; Cell Signaling Technology), Akt (1:1000; Cell Signaling Technology), or β-tubulin (1:1000; Sigma) overnight at 4°C. The membrane was then rinsed with TBST and incubated with an HRP-labeled secondary antibody (1:5000; Bioworld, USA) at 37°C for 45 min, and the signals were detected with ECL reagent. Specific bands were detected with an electrochemiluminescence system (Bio-Rad, Hercules, CA, USA), imaged using the ChemiDoc XRS gel imaging system (Bio-Rad), and quantified by densitometry (ImageJ, National Institutes of Health, Bethesda, MD, USA). All the experiments were performed in triplicate.

### Scanning electron microscopy (SEM)

For the SEM analysis of the pericyte shape and activity, the samples were fixed in glutaraldehyde, postfixed for 2 h in 4% osmium tetroxide and dehydrated via a graded series of ethanol solutions. Continuous cross-sections were obtained using the freeze-fracture method. Pretreated samples were dried in a critical point drier (Hitachi, Tokyo, Japan) and coated with gold (ion sputter coating method) for observation using a SU8100 scanning electron microscope (Hitachi).

### Statistical analysis

The analyses were performed using Statistical Program for Social Science (SPSS) version 22.0. The data are reported as the means ± standard deviations (SDs). For analysis of the background data of the included patients, the measurement data were analyzed using one-way ANOVA tests, and the enumeration data were analyzed with Fisher’s exact tests. The MWM results were analyzed by repeated-measures one-way analysis of variance (ANOVA). The immunofluorescence, qRT-PCR, western blot, MWM test and MRI-ASL results were evaluated by two-tailed unpaired Student’s t-test. Differences were considered significant if P < 0. 05 or very significant if P < 0.01.
